# Maladaptive behaviours of maternal orphans in high schools of Tshwane North of Gauteng, South Africa

**DOI:** 10.4102/phcfm.v15i1.3887

**Published:** 2023-10-16

**Authors:** Thembi V. Simbeni, Mathildah M. Mokgatle

**Affiliations:** 1 Sub-department of Health Systems Management and Policy, Department of Public Health, School of Health Care Sciences, Sefako Makgatho Health Sciences University, Pretoria, South Africa; 2 Sub-department of Epidemiology and Biostatistics, Department of Public Health, School of Health Care Sciences, Sefako Makgatho Health Sciences University, Pretoria, South Africa

**Keywords:** maladaptive behaviour, orphaned adolescents, maternal orphans, Tshwane North, Gauteng province

## Abstract

**Background:**

Some orphaned adolescents find it difficult to cope and adjust to the loss of a mother. Studies to explore specific adjustment challenges experienced by this vulnerable group, are necessitated by the growing need to inform support services for orphans.

**Aim:**

This study sought to explore maladaptive behaviours among adolescent maternal orphans.

**Setting:**

Participants were recruited from the Tshwane North secondary schools of Gauteng province in South Africa.

**Methods:**

A qualitative exploratory design was employed; maternal adolescent orphans were purposively selected and included in a one-on-one qualitative enquiry. Twenty-five participants were included in the study. Data were analysed thematically using NVivo12.

**Results:**

Emerged themes were: negative thoughts such as suicidal ideation, negative perception of self; silence coded as ‘keep life matters private and hide personal feelings’; having psychosocial problems reported as anger, fighting, shouting, crying, short temper; engaging in risky behaviours in the form of smoking and alcohol use and unsafe termination of pregnancy; social withdrawal by self-isolation and being afraid of people.

**Conclusion:**

Whole school peer interaction groups could address the functional problems of social ability and silence. Skills development programmes, and other activities that enhance constructive use of free time, instil hope and build self-esteem are recommended.

**Contribution:**

The findings of this study serve as a basis to inform interventions that are geared towards supporting adolescent orphans through the school health teams, as one of the domains of the re-engineering of South Africa’s primary health care system.

## Introduction

The adversity of losing a mother results in devastating consequences in the lives of most adolescents. In most instances, the result is psychosocial problems that impact negatively on the mental health of orphans, their relationships with family members and peers and learning at school and engagement in pleasurable activities with peers.^1,2^ The common psychological problems that negatively impact on adjustment and functioning include, hopelessness, anger, short temper and irritability, among others.^1,3,4^

Studies revealed that some orphaned adolescents prefer to be silent about things that distress them, including the aspect of having lost parents, to avoid being treated differently by peers.^5,6,7^ Some orphaned adolescents experience feelings of hopelessness, suicidal ideation, social withdrawal and poor self-concept.^4,5,8^ Their troubled emotions can strain relationships with guardians, educators and fellow orphans who may not recognise that they are depressed.^4^ Emotional and behavioural problems negatively impact on their home life, learning at school, free time activities and their peer relationships.^1^

Additionally, studies show that being an orphan is associated with the onset of sexual activity and, for some it is 13 years or younger.^9,10^ The reasons for orphans engaging in sex differ and can be in exchange for something or for survival, in reaction to distress or high levels of depression, insufficient or lack of adult care, love and supervision, for seeking love, approval or belonging from peers that may then not be in their lives and for other different reasons.^9,10^ Studies show that maternal or double orphans are more likely to have used alcohol or drugs for pleasure.^11,12^

Previous studies found a strong association between substance use and sexual risk behaviours; this included alcohol use, drug use and smoking being associated with sexual risk actions in general.^13,14^ Research work on orphans in South Africa paid more attention on describing their physical needs and living arrangements and other external factors that influence their wellbeing.^15^ There is paucity of literature on psychological adjustment problems among maternal orphaned adolescents. The objective of this study is to explore maladaptive behaviours among adolescent maternal orphans in Tshwane North of Gauteng province. Findings of this study will serve as a basis to recommend ways to support adolescent orphans in adjusting appropriately to the circumstance of having lost a mother.

## Research methods and design

### Study design

A qualitative exploratory design was used to gain an in-depth understanding of behaviours that make maternal orphans thrive or not thrive in their daily lives. This article presents the maladaptive behaviours among orphaned adolescent subjects.

### Study population and setting

The study was conducted among maternal orphans attending four secondary schools, in the North of Tshwane, which is based in Gauteng province. A full description of the population, and the setting are described in detail in a publication elsewhere.^16^

### Sampling and sample size

The authors purposively selected maternal adolescent orphans who had the highest depression scores for inclusion in the qualitative sample.^16^ The researcher allowed data saturation to determine the sample size, which was 25. Variables such as gender, duration of orphanhood, orphan type and location were considered when selecting the sample.

### Data-collection instruments

The socio-demographic data collection comprised section A of the data-collection tool. This was followed by section B of the data-collection tool comprising the interview guide. The socio-demographic data collection form was a brief researcher-administered survey that contained questions on the following: age, gender, school grade, relationship with primary caregiver, primary caregiver employed, receiving a social grant, type of social grant received, primary caregiver’s occupation, number of adults in the household, number of siblings and number of other children in the household. An English-language researcher developed an unstructured interview guide with open-ended questions which was used to collect data. Questions in the interview guide were informed by concepts of the Bronfenbrenner’s ecological systems theory. Questions were only informed by the individual aspect of the microsystem of the Bronfenbrenner’s ecological systems theory. The interview guide was translated to Setswana for the interviews.

Some of the questions in the in-depth interview guide were:

- Explain whether you have someone to talk to at home.
- Explain whether you have someone to talk to at school.
- What do you do when you are sad about something at home?
- Explain whether there is something that makes you happy at home.
- Explain whether there is something that makes you happy at school.
- What do you do when you are bothered by something when at school?
- What do you do when you are bothered by something at home?
- Describe how you spend your free time at home.
- As a child who has lost a mother, explain what goes on in your mind when you are sitting alone.

### Data-collection method and procedure

All in-depth interviews were conducted by two people, a researcher and one trained and experienced research assistant. Data collection occurred in a private room in the school premises. The school setting proved to be inappropriate for in-depth interviews as there was disturbance as a result of noise from classes next to the room where the data were being collected. The authors therefore decided to conduct the in-depth interviews after school hours, when most children had left the premises. This was to allow the one-on-one in-depth interviews to occur in a noise-free environment, so that the recorded data would be clearly audible with no noise in the background. The interviews were conducted in Setswana, a predominant language in Tshwane North District of Gauteng province. The use of Setswana language made it easier for the study participants to provide in-depth explanations and descriptions in response to the questions asked by us.

A digital voice recorder was used to record all the interviews. To help obtain an in-depth understanding of the participants’ self-reports, we ensured asking questions in a manner that elicited explanations and descriptions, by asking probing questions where necessary, by asking for clarification where responses were not clear and by allowing participants to fully respond to the questions asked without interrupting them.

Only one in-depth interview was conducted in a day, and three interviews in a week with a week’s break in between. This allowed the researcher time to listen to the audio-recorded interviews with the co-researcher, to identify shortcomings in the phrasing of certain questions and to rephrase questions or adjust the tool accordingly and subsequently improve the quality of the data, as the data collection continued. Data collection occurred for a period of 3 months.

### Data analysis

Precise transcription of the audio-recorded data was conducted; the Setswana transcribed data were translated to the English language verbatim. The thematic analysis approach was used to analyse the data. We read the transcripts several times to get familiarised with the data and to identify initial emerging codes. A codebook with corresponding definitions was developed from the first five transcripts. All transcripts were then imported into Nvivo12 software and codes were applied to all the transcripts. Themes were identified and used to present the findings. Data analysis was undertaken in close collaboration with the co-researcher, who is an experienced researcher and took up the role of an independent coder.

### Trustworthiness

The authors trained the research assistant in transcribing the qualitative data, and further ensured accurate transcription and translation of the data by verifying the translation and transcription conducted by the research assistant. Translation and transcription were verified for accuracy by listening to the first three pieces of audio recorded data and reading the transcribed translated data. Errors were shown to the research assistant early during transcription and translation to ensure accurate representation of the participant’s words. Peer debriefing sessions were conducted early in the data-collection phase to identify weakness and rephrase certain questions on the tool to allow collection of rich data and to verify the Nvivo data coding and analysis. Prolonged engagement in the field took place, with us interviewing only three participants per week. This was to ensure initiating coding and identifying themes as data collection continued, and it enabled us to obtain all the necessary data from the field to adequately inform the study.

### Ethical considerations and recruitment

Ethical approval of the study was obtained from the Sefako Makgatho Health Sciences University Research Ethics Committee (the SMUREC) and SMUREC/H/241/2017: PG was the project number. Permission was also sought from the Provincial Department of Basic Education and from the relevant schools. When the school leadership had given the researcher permission to conduct the study, one teacher was allocated to us in each school to assist us in identifying potential study participants and to give us lists of orphans from the orphan statistics records of the school. From the orphan statistics records of the schools, we developed a sampling frame by purposively selecting participants who had the highest CDI 2 score and possessed characteristics of interest that will enable them to inform the study. For schools that did not keep records of orphans, researchers had to identify orphaned children by administering the socio-demographic part of the tool to all children in all classes of the school with the assistance of the teacher. The socio-demographic form was self-administered, and data from the forms were used to identify maternal and double orphans who met the inclusion criteria. Identifying potential study participants in this manner allowed them the confidentiality of their personal information, as some children did not wish to reveal their orphan status to fellow learners at school.

Identified potential study participants were recruited in a separate private room. When recruiting the study participants, details about the study and the purpose of the study were explained. Potential study participants were given an opportunity to ask questions or for any clarification regarding the study. It was explained to the participants that participation in the study was voluntary and that if at any time they decided to withdraw from the study, their decision would be respected. Those who declined were not involved in the study, and they were informed that their decision would not in any way impact on their relationship with their teachers or their school performance. Potential study participants who were willing to participate were given a guardian’s consent form containing all the information pertaining to the study, for the guardian to sign at home. The potential study participants brought back the signed guardian’s consent form on the next day, and they themselves also signed a written informed assent form. Participants were informed that participation is voluntary and that they can withdraw from the interviews or the study at any time. Confidentiality was ensured and fictitious names were used to conceal the identity of participants and data were stored safely. The researcher and the research assistant allowed participants to give themselves fictitious names.

## Results

### Socio-demographic characteristics of the participants

#### Socio-demographic characteristics of this study participants are outlined in Table 1

All 25 participants were aged between 13 and 17 years; 17 were female, all had lost a mother and 16 were double orphans (see Table 1). They were all living in households within communities. A number of them (*n* = 11) were living in extended families with the grandmother being the guardian. Two were caring for themselves and heading a home, where they lived with younger siblings. Furthermore, approximately half (*n* = 12) of the participants were living with members of their families other than their grandmothers and, this included either living with a father, aunt, sister or uncle and, among them only one was living with a man not related to him. More than half (*n* = 17) of the participants were living with an unemployed caregiver while few of them (*n* = 8) had employed caregivers. Half of the participants were not receiving a social grant and their reasons for not receiving it varied, from still organising to obtain a birth certificate, to the grant being stopped by the previous guardian when some of them moved to live with the current guardian. Others were maternal orphans whose fathers were working and did not qualify to obtain the social grant although not living with the father. Some participants were living in urban townships and others in a semi-rural area of Tshwane North district of Gauteng province.

**TABLE 1:** Socio-demographic characteristics of the study participants (*N* = 25).

Characteristics *n* = 25	Number
**Age**	
13–17	25
**Gender**	
Male	8
Female	17
**Who do they live with**	
Alone (heading home)	2
Grandparents	11
Siblings (Sister)	3
Guardians (Aunt or uncle)	6
Father	3
**Orphan type**	
Maternal orphan	9
Double orphan	16
**Receives a social grant**	
Yes	13
No	12
**If you are receiving a social grant, tick the type of a grant**	
Foster care grant	8
Child support grant	5
Not getting a grant	12
**Caregiver employed**	
Yes	8
No	17

### Findings from the interviews

Table 2 shows the theme and subthemes from the analysis of the data in this study. The table shows that participants had psycho-social problems as a theme and the following five subthemes that describe the maladaptive behaviours of the participants: (1) negative thoughts, (2) silent, (3) emotional problems, (4) engage in risky behaviours and (5) social withdrawal. Table 2 presents the codes for these behaviours.

**TABLE 2:** Themes and codes aligned to maladaptive behaviours.

Theme: Psycho-social problems	
Subthemes	Codes
Negative thoughts	Suicidal ideation
	Suicidal attempt
	Hopelessness about life and the future
	Negative perception of the self
Silent	Do not consult teachers for clarity regarding schoolwork
	Keep life matters private
	Hide personal feelings
Emotional problems	Anger, fighting, shouting, crying, short temper
Engage in risky behaviour	Use of addictive substances
	Smoking and alcohol use
	Early sexual debut
	Unsafe termination of pregnancy.
	Nighttime taverns and streets
Social withdrawal	Avoid contact with people
	Self-isolation
	Shy, afraid of people
	Uncomfortable around people

#### Psycho-social problems

The theme psychosocial problems emerged with the further discussed subthemes that show maladaptive adjustment to the circumstance of parental loss. The maladaptive behaviours include vulnerability, a lack of resilience and a lack of personal protective behaviour that puts the orphaned in a position not to seek help when faced with problems.

### Negative thoughts

The narratives show that orphans in this study had thoughts of ending their lives and, some of them attempted to end their lives. They experienced feelings of hopelessness about their future. They had a poor self-concept in that, they had a negative view about themselves as individuals and about their physical image.

‘I feel like killing myself, I then tell my family “I am tired of this life … it’s like you can kill me or I wish my mother had aborted me, spare me from seeing this life”.’ (Tebogo, 17-year-old, female, double orphan)

Morongia explained her friend’s reaction to her numerous attempts to commit suicide:

‘I tried to kill myself so many times at some point Khothatso was crying, telling me that “I shouldn’t try it again, I sometimes think about doing it but then not do it, I don’t know what happened that I don’t do it but it comes frequently”.’ (Morongoa, 16-year-old, female)

‘Most of the time when I am alone, I just think of how I am going to die, things like that. I never think of my future.’ (Lerato, 15-year-old, female)

‘I just don’t know how to put it, I am a straat mate I have no dignity.’ (Mapula, 17-year-old, female)

‘Sometimes I feel like there’s something missing when I am dressed, I feel like I am messed up.’ (Petunia, 17-year-old, female)

‘When I am with people, I have thoughts that they are saying “what is this thing without a mother doing here” but these are my thoughts.’ (Tshepang, 15-year-old, female)

### Silent

The narratives revealed that orphans are silent about things that bother them at home and at school. They reported that when they are hurt by peers at school, they ignore them. They keep quiet to keep their personal matters private at school and are unable to approach teachers at school when they do not understand some of their schoolwork. Most of them mentioned they often do not reveal their true feelings and opinions and, some of them mentioned that they have no one to talk to at home.

‘I just ignore them and look at them because if I start arguing they will say some mean words that will hurt me and I have anger issues so I will want to physically fight with them.’ (Katlego, 17-year-old, female)

‘Maybe it’s the thing of trying to keep my life private, when I am at school, I do not want people knowing my problems.’ (Lerato, 15-year-old, female)

‘There are some subjects that I don’t understand, maybe like consumer. I need someone to explain to me and there is nobody. I am not used to going to the teachers, I don’t go to the teachers.’ (Kaiser, 16-year-old, male)

‘I hide my painful personal feelings. No matter how angry I am, I would just laugh so you won’t tell that I don’t like this and that.’ (Tshepang, 15-year-old, female)

‘It’s a lot of things but I only talk to them about things related to school. But personal things I do not tell them, even when I need something I don’t tell them. They will be the ones to ask if I need anything.’ (Bokang, 15-year-old, male)

‘I don’t talk to anyone at home unless people outside, but I don’t have anyone to talk to at home. I cry then sleep.’ (Ditshego, 17-year-old, female)

### Emotional problems

Majority of orphans in this study talked of having emotional difficulties such as anger, irritability, sadness, short temper and crying. Their emotional difficulties manifest in fighting and shouting at their peers, anger outbursts, being too quick to cry and sadness as the following quotes illustrate:

‘I think that is why they don’t come to me, because they know that if others come to me, I will not respond in a polite way to them. You find that they came to me or want to be happy with me but then I won’t entertain them.’ (Bokang, 15-year-old, male)

‘When I say I don’t like people it is because people, they turned me into this, those who raped me, they led me to doing abortions. When I see a person, I feel like …, especially a man, I do not want them, I just get angry. I feel like he is the one who did this to me, all of it I feel like, when I see him, I feel like … even when it is my friend … I feel as if he is the one who did this to me. I then get angry and say “hai leave it, hai!!!”.’ (Tebogo, 17-year-old, female, double orphan)

‘Sometimes when we are together and they talk about their parents, I get angry and shout at them, I am always fighting with them.’ (Thandazo, 17-year-old, female)

‘I am a person who is too quick to cry, I’m too emotional even just now the way I am now I am emotional. Someone can just say something bad then I will quickly get angry even the smallest of things I will overreact.’ (Pertunia, 17-year-old, female)

‘At times I ask myself why I am not bubbly like other kids, like I don’t laugh at things they laugh at, I am too still.’ (Morongoa, 16-year-old, female)

### Engage in risky behaviours

Narratives revealed that some orphaned adolescents engage in risky behaviours which include roaming around the taverns and streets at night, associating with strange men in exchange for alcohol and getting rides from strange people at night. Data further show that some of them use addictive substances, experience early sexual debut and, one of them reported to have had an unsafe termination of her pregnancy.

‘At the clinic they told me this child [*pregnancy*] it’s a child there is no way I can kill “it”. But I knew that I have connections if I can connect my things well and be able to get the money and do what I can to get pills. So, I did the abortion. I did the abortion at fourteen weeks, five days.’ (Tebogo, 17-year-old, female)

‘I ask myself why do I have to make such decisions like having sex with boys, my friend is still a virgin and I ask myself why do I have sex when I am still so young.’ (Katlego, 17-year-old, female)

‘In most cases we drink Lean, Lean is a cough syrup mixed with cold drink.’ (Mapula, 17-year-old, female)

‘We smoke mam. Eish mam we smoke Nyaope.’ (Themba, 17-year-old, male)

‘I started smoking after my mother passed away, she was also smoking because she wasn’t treated in a good way. So, when my mother passed away, I thought a cigarette will reduce my stress, so I started smoking.’ (Thandazo, 17-year-old, female)

‘I started drinking, smoked, hanged around bad people then I started isolating myself, I changed my behaviour.’ (Mapula, 17-year-old, female)

‘At 02:00 each of us goes back home, the one who has been chased out of home, she goes with boys. She meets people, isn’t it that in the streets at night people call you, they call you, we know that we let her get the ride.’ (Tebogo, 17-year-old, female)

‘When he has proposed love to you and you are there with him, he buys beer for you. When you finish to drink … when you feel that you are now drunk, and you can’t even control yourself we runway from them and go home. We call each other and run away that is the life we were living.’ (Tebogo, 16-year-old, male)

### Social withdrawal

Majority of the study participants acknowledge the fact that following the loss of their mother, they started feeling ‘uncomfortable’ around people, they avoid interacting with peers, teachers and other people at home and at school. They avoid interaction with peers in pleasurable activities and, are also withdrawn in class at school. Most of them reported that they are naturally quiet, and their peers are afraid of approaching them, so they spend most their time alone. A few of them reported not knowing how to engage in a conversation in that they often do not know what to say.

‘Like going out to movies, so when they say, “let’s go watch a movie” I tell them that I am at home and do not want to do anything.’ (Bokang, 15-year-old, male)

‘I was no longer the same person; I stopped being comfortable around people.’ (Tshepang, 15-year-old, female)

‘I eat outside under the tree alone when I finish eating, others would come and sit under the shade with me then I leave and go back to class and just sit there on my own.’ (Kgosi, 15-year-old, male)

‘I struggle to socialise with other people maybe it’s the way that I am because others think that I don’t like people are afraid of approaching me and I am just like that, I am a quiet person, but I do talk on some days. I have time to talk ad to keep quiet, so I feel as if I struggle to communicate and to socialise more and go on outings with other kids.’ (Thato, 16-year-old, male)

‘That’s when I stopped having friends at home.’ (Kagiso, 16-year-old, male)

‘I don’t know how to talk to people, in most cases to make friends you have to engage in conversations right? Where you talk, and for me it’s difficult because I don’t know what to say to them.’ (Mapula, 17-year-old, female)

‘Teachers are surprised, you find them asking “who is Busisiwe?” while I’m silent I never participate I feel like to raise your hand and talk I am scared of people. I am scared of people, I don’t participate they don’t know me. You will hear them asking “who is Neo?”.’ (Neo, 15-year-old, female)

‘I used to enjoy being around people, I loved hanging around with other people. I loved to play. From there, I started being alone. Even in class, one teacher became surprised she called my aunt she said, “what happened to this child?”.’ (Calvin, 16-year-old, male)

## Discussion

This study explored maladaptive behaviours among orphaned adolescents who were living with families in urban townships and semi-rural areas of Tshwane North district, in Gauteng province of South Africa. All of them were in junior secondary and high schools. Most of them were being raised by their grandmothers, some by either maternal or paternal aunt or uncles. Few of them lived with their fathers and with their sisters, and a couple of them were living with siblings and headed their homes.

The authors found that because of the prolonged difficulty in coping with or in adjusting to the circumstance of the loss of a mother, participants in this study experienced negative thoughts such as suicidal ideation, suicidal attempts and hopelessness about the future. These finding are consistent with other studies that revealed that some orphaned adolescents experience feelings of hopelessness, suicidal ideation, social withdrawal and poor self-concept.^4,5,8^

Low self-esteem, anger, sadness, irritability and short temper were some of the psychological problems that were reported by participants in this study. This study data show that these psychological problems emanated from their experienced traumatic events such as the death of a parent itself, and for some from their experience of physical, verbal or sexual abuse by people in their life context. Similar findings were reported elsewhere.^1,17,18^ Their troubled emotions lead to difficulties in forming a wide network of friends and difficulties in relating with other people in their lives who may not be aware of these emotional problems. Similar findings have been reported elsewhere.^4,19,20^

Most participants in this study reported that when they were at home, they were silent about things that bothered them. They attributed this behaviour to not having a close, open relationship with their caregivers. Additionally, because they preferred to keep their personal lives including their orphanhood status private at school and were also shy to approach teachers when they did not understand some of their schoolwork, they did not express themselves at school when they were bothered about something. This means that this group of adolescents is not sourcing this crucial caregiver emotional support, as well as support from teachers and peers and is therefore predisposed to the risk of mental health problems. Similar findings regarding silence about their feelings were reported in a study conducted by Ntuli that revealed that higher levels of emotional support from the caregiver were associated with fewer mental health problems among orphaned adolescents.^5^

The use of addictive substances, unsafe abortions and unprotected sex, roaming the streets and taverns at night, accepting car rides from strange men at night, receiving alcohol offers from strange men and running away were behaviours that were reported by some of the participants in this study. These behaviours may result from the fact that most adolescents who reported these behaviours were double orphans, and some had no stable homes, which meant that there was no consistent stable supervision from an adult, while others had caregivers who did not pay much attention to them. The loss of a parent or both parents often leaves the orphan with altered emotional states and physical resources, as well as a lack of attention and care from family adults, which predisposes them to risky behaviours.^12^ Similar findings regarding risky sexual behaviours of orphans and the reasons for the behaviour were reported elsewhere.^9,10^

A common statement made by the participants in this study was that following the loss of their mother, they avoided contact with people and had feelings of wanting to be alone at home and at school. Social withdrawal is a symptom of depression. Interpersonal problems present themselves as difficulty in forming and maintaining a wide network of friendships which can be because of avoiding contact with people, low self-esteem and mood problems. Studies have shown that children who are depressed are more often withdrawn from others and so may acquire insufficient interpersonal skills.^4^

## Conclusion and recommendations

Some of the participants in this study displayed maladaptive behaviours following the loss of their mother. The reported maladaptive behaviours included experiencing negative thoughts such as suicidal ideation, suicidal attempts and hopelessness, negative views about oneself, engagement in risky behaviours such as abuse of substances, early sexual debut and unprotected sex and unsafe abortions. To address the above reported maladaptive behaviours, existing community programmes, in collaboration with the social development department including community-based non-governmental organisations (NGOs) and churches, can organise music groups, skills development programmes, creative arts and other economical indigenous activities to enhance constructive use of free time, develop and instil hope and self-esteem among this group of adolescents and other adolescents in general.^21,22^

To address the reported social withdrawal, difficulties in relating to people and maintaining silence about issues that bother them, it is recommended that the Department of Basic Education implement whole-school peer interaction groups. Whole-school peer interaction groups comprise a public health intervention that promotes school community relationships and social and emotional skills which contribute towards positive youth development.^23^

The peer groups can be facilitated by the life orientation educator, to enhance social interaction, social skills development and talking about common experiences, problems and possible solutions.
